# Clinical Relevance of New World Health Organization Classification System for Pituitary Adenomas: A Validation Study With 2-Year Experience

**DOI:** 10.3389/fonc.2021.739290

**Published:** 2021-09-13

**Authors:** Seung Woo Hong, Se Hoon Kim, Seung Hoon Lim, Eun Jig Lee, Sun Ho Kim, Cheol Ryong Ku, Eui Hyun Kim

**Affiliations:** ^1^Department of Neurosurgery, Yonsei University College of Medicine, Seoul, South Korea; ^2^Department of Pathology, Yonsei University College of Medicine, Seoul, South Korea; ^3^Pituitary Tumor Center, Severance Hospital, Seoul, South Korea; ^4^Yonsei Endocrine Research Institute, Yonsei University College of Medicine, Seoul, South Korea; ^5^Division of Endocrinology and Metabolism, Department of Internal Medicine, Yonsei University College of Medicine, Seoul, South Korea; ^6^Department of Neurosurgery, Ewha Woman’s University College of Medicine, Seoul, South Korea

**Keywords:** immunohistochemistry, pituitary adenoma, pituitary hormone, transcription factor, WHO classification

## Abstract

**Background:**

The new World Health Organization (WHO) classification system proposed a cell lineage-based classification scheme for pituitary adenomas in which transcription factors (TFs) play a major role as key classifiers. We aimed to evaluate clinical relevance of the new classification system in a clinical setting.

**Methods:**

TF staining was retrospectively performed for 153 clinically and histologically well characterized pituitary adenomas. Then, 484 pituitary adenomas were prospectively stained for TFs and then for relevant pituitary hormones. TF and hormone stain-based diagnoses were compared, and differences in clinical manifestations were evaluated.

**Results:**

The accuracies of antibodies for three TFs were successfully validated and had an overall matching rate was 89.6%. We identified 50 (10.4%) cases with discrepancies between TF and pituitary hormone stains. Gonadotroph adenomas lacking follicle-stimulating hormone and luteinizing hormone stains account for most discrepancies. Null cell adenomas may be more prevalent than reported and may be clinically more aggressive than gonadotroph adenomas.

**Conclusion:**

The new WHO classification is mostly well matched with the traditional classification. However, until the new classification is further validated and interpreted in the context of long-term clinical outcomes, routine histological examination should include full slate of immunostains for pituitary hormones as well as TFs.

## Introduction

Pituitary adenomas are neuroendocrine tumors in the anterior pituitary gland. They are traditionally classified based on their hormonal activity as non-functioning and endocrine-active tumors. Histopathological examination is important to confirm the diagnosis by validation of positive immunohistochemistry (IHC) for relevant pituitary hormones. The fourth edition of the World Health Organization (WHO) classification of endocrine tumors was published in 2017 (1). One of the major changes in the new scheme is the cell lineage-based classification of pituitary adenomas characterized by lineage-specific transcription factors (TFs). All pituitary adenomas are divided into the three following lineages: lactotroph, somatotroph, and thyrotroph belong to PIT-1 (pituitary-specific TF 1), corticotroph belongs to T-PIT (pituitary cell-restricted factor), and gonadotroph belongs to SF-1 (steroidogenic factor 1). Tumors negative for all three TFs are considered as true null cell adenomas.

In September 2018, we started to provide pathological diagnoses for all pituitary adenomas surgically removed in our institution based on the new WHO classification system. Based on our 2-year experience, we aimed to evaluate the clinical relevance of new classification system and discrepancies between pituitary hormone-based and TF-based diagnoses.

## Materials and Methods

This study was conducted in accordance with the Declaration of Helsinki and approved by the local Institutional Review Board. The IHC antibodies used for TFs and pituitary hormones are listed in **Table 1**. Every single sample was examined by two experienced neuropathologists. The hormone-positivity was described as focal/diffuse and weak/strong. For the stains for TFs, roughly 5% of cutoff was adopted. We considered cases with even very week stains for TFs in the nucleus as positive.

**Table 1:** Antibodies used for immunohistochemistry for transcription factors and pituitary hormones.

	Company	Clone	Dilution factor	Machine	Expression pattern	Antigen retrieval	Antibody primary
	Transcription factors						
PIT-1	Novus Biologicals	NBP1-92273	1/500	Ventana, BenchMark XT	Nuclear	Conventional	BenchMark XT instrument, 32 minutes, 37°C
T-PIT	Atlas Antibodies	AMAb91409	1/1000	Dako, Omnis	Nuclear	Low pH, 30 minutes	Omnis instrument, 20 minutes, 32°C
SF-1	Perseus Proteomics Inc.	PP-N1665-0C	1/400	Dako, Omnis	Nuclear	Low pH, 30 minutes	Omnis instrument, 20 minutes, 32°C
	Pituitary hormones						
GH	DAKO, Agilent	A0570	1/400	Ventana, BenchMark XT	Cytoplasmic	Not required	BenchMark XT instrument, 32 minutes, 37°C
PRL	DAKO, Agilent	A0569	1/300	Ventana, BenchMark XT	Cytoplasmic	Not required	BenchMark XT instrument, 32 minutes, 37°C
TSH	DAKO, Agilent	M3503	1/50	Ventana, BenchMark XT	Cytoplasmic	Not required	BenchMark XT instrument, 32 minutes, 37°C
ACTH	DAKO, Agilent	M3501	1/200	Ventana, BenchMark XT	Cytoplasmic	Not required	BenchMark XT instrument, 32 minutes, 37°C
LH	DAKO, Agilent	M3502	1/50	Ventana, BenchMark XT	Cytoplasmic	Not required	BenchMark XT instrument, 32 minutes, 37°C
FSH	DAKO, Agilent	M3504	1/50	Ventana, BenchMark XT	Cytoplasmic	Not required	BenchMark XT instrument, 32 minutes, 37°C

ACTH, adrenocorticotropic hormone; FSH, follicle stimulating hormone; GH, growth hormone; LH, luteinizing hormone; PIT-1, pituitary specific transcription factor 1; PRL, prolactin; SF-1, steroidogenic factor 1; T-PIT, pituitary cell restricted factor; TSH, thyroid stimulating hormone.

### Retrospective Validation

Before September 2018, our routine pathological examination for pituitary adenomas included IHC for all anterior pituitary hormones: adrenocorticotropic hormone (ACTH), growth hormone (GH), prolactin (PRL), thyroid-stimulating hormone (TSH), luteinizing hormone (LH), and follicle-stimulating hormone (FSH). We retrospectively selected 49 non-functioning pituitary adenomas including 27 null cell tumors and 22 gonadotroph adenomas. None of these patients presented any symptoms suggesting hormonal excess. We then identified 104 endocrine-active pituitary adenomas: 36 GH-secreting adenomas, 28 prolactinomas, 21 TSH-secreting adenomas, and 19 ACTH-secreting adenomas. For all 104 patients with endocrine-active pituitary adenomas, their hormonal excess was well matched with laboratory tests and clinical symptoms. Acromegaly was defined when the nadir serum GH level after an 75g oral glucose tolerance test was less than 1.0 ng/mL with elevation of serum insulin-like growth factor-1 adjusted for age and sex. The diagnosis of Cushing’s disease was established on the basis of clinical features and the results of biochemical tests including 24 h urinary free cortisol excretion, low- and/or high-dose dexamethasone suppression test. And bilateral inferior petrosal sinus sampling was performed in all cases. The diagnosis of TSH-secreting pituitary adenoma was made based on serum TSH, free thyroxine, triiodothyronine levels together with the radio of free alpha subunit and TSH, which was later confirmed by postoperative normalization of TSH. A T3 suppression test was performed when it was unclear whether the patient had a TSH-secreting pituitary adenoma or a non-functioning pituitary adenoma in the presence of secondary hyperthyroidism. IHC of three TFs was then performed for cell-lineage classification. The classifications based on pituitary hormones and TFs were compared.

### Prospective Validation

**Figure 1:**
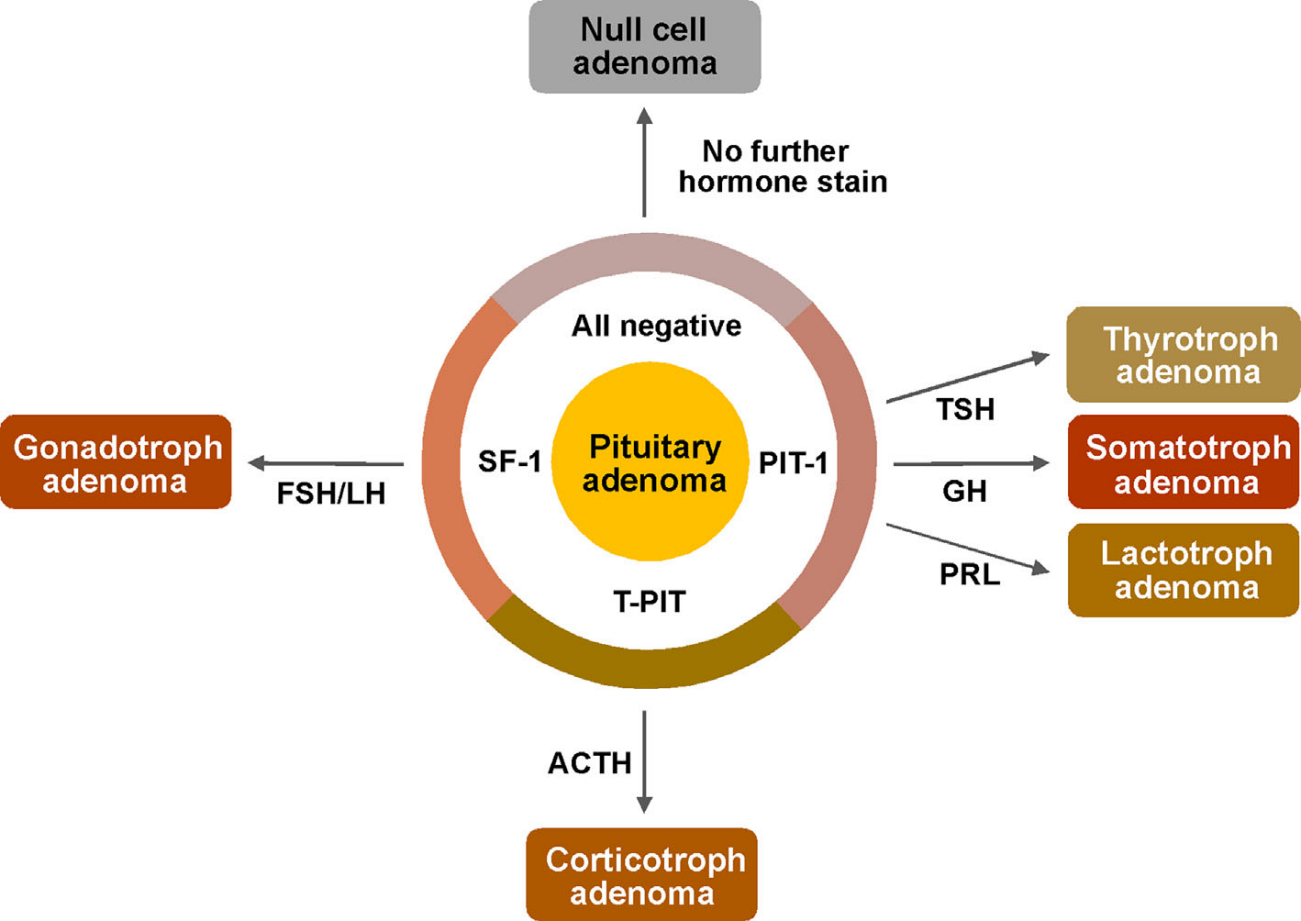
Pathological examination protocol for pituitary adenomas based on the new WHO classification. First, immunostains of transcriptions factors (PIT-1, T-PIT and SF-1) were performed for determination of cell lineage. And then, following stains were performed only for possibly associated hormones based on the result of TF stains; GH, PRL and TSH for PIT-1 positive adenomas, only ACTH for T-PIT positive adenomas and LH, FSH for SF-1 positive adenomas. ACTH, adrenocorticotropic hormone; FSH, follicle stimulating hormone; GH, growth hormone; LH, luteinizing hormone; PIT-1, pituitary specific transcription factor 1; PRL, prolactin; SF-1, steroidogenic factor 1; T-PIT, pituitary cell restricted factor; TSH, thyroid stimulating hormone.

Since September 2018, the routine pathological examination protocol for pituitary adenomas was updated based on the new WHO classification (**Figure 1**). Informed consent was obtained from enrolled patients. We first performed IHC for three TFs: PIT-1, T-PIT, and SF-1. Then, IHC was performed for possibly associated hormones based on the result of TF stains: GH, PRL, and TSH for PIT-1 adenomas; only ACTH for T-PIT adenomas; and LH and FSH for SF-1 adenomas. When the hormone stain results were not matched to TF stains, the tests were repeated and IHC was performed for all pituitary hormones. For all discordant cases, IHC for TF stains and hormone stains were repeated to confirm the results. Cases with incomplete study and with pituitary apoplexy that prevented reliable IHC were excluded from this study. A total of 484 patients who underwent surgical resection for their pituitary adenomas by two neurosurgeons from September 2018 to August 2020 were included in this analysis. First, hormone and TF stains were compared to determine whether they were well matched. For patients with concordant results, histological and clinical diagnoses were compared with subgroup analysis of their tumor nature.

### Statistical Analysis

We performed *t*-tests and chi-square tests to identify statistically significant differences. All analyses were performed using IBM SPSS Statistics (version 20.0; IBM, Armonk, NY, USA), and *P* < 0.05 was considered statistically significant.

## Results

### Retrospective Validation

The results of our retrospective analysis are summarized in **Table 2**. For 153 cases of pituitary adenomas previously operated in our institution based on the traditional classification scheme, we performed IHC for three TFs and compared the findings. The results were consistent in 149 (97.4%) cases. We identified only five cases with discrepancies between hormonal and TF stains. One patient with overt Cushingoid features and positive ACTH stain was negative for all TFs. There were four endocrine-inactive pituitary adenomas with negative stains for all pituitary hormones; a positive stain for T-PIT was observed in one patient and SF-1 in three patients.

**Table 2:** Retrospective comparison of immunohistochemistry between pituitary hormones and transcription factors.

Pituitary hormone stain	Number of cases examined	Number of cases with matched TFs	Details on mismatched cases
Single ACTH positive	19	18 (94.7%)	Negative for all TFs
Single PRL positive	28	28 (100%)	
Single GH positive	36	36 (100%)	
Single TSH positive	21	21 (100%)	
Positive for FSH and/or LH	22	22 (100%)	
All negative	27	23 (85.2%)	Positive for T-PIT in 1 and SF-1 in 3
Total	153	148 (96.7%)	

ACTH, adrenocorticotropic hormone; FSH, follicle stimulating hormone; GH, growth hormone; LH, luteinizing hormone; PRL, prolactin; SF-1, steroidogenic factor 1; TF, transcription factor; T-PIT, pituitary cell restricted factor; TSH, thyroid stimulating hormone.

### Prospective Validation

The cohort included 303 endocrine-inactive tumors and 181 endocrine-active tumors including 86 GH-secreting pituitary adenomas, 57 prolactinomas, 36 ACTH-secreting pituitary adenomas, 2 TSH-secreting adenomas. While prolactinoma is known to be the most common type of pituitary adenomas, the prevalence of prolactinomas is possibly underestimated in our surgical series because majority of them were treated with dopamine agonists without pathological diagnosis. Also, compared to other series in the literature, much more patients with endocrine-inactive pituitary adenomas (62.6%) were enrolled to our study.

### Comparison Between TF and Pituitary Hormone Stains

**Figure 2:**
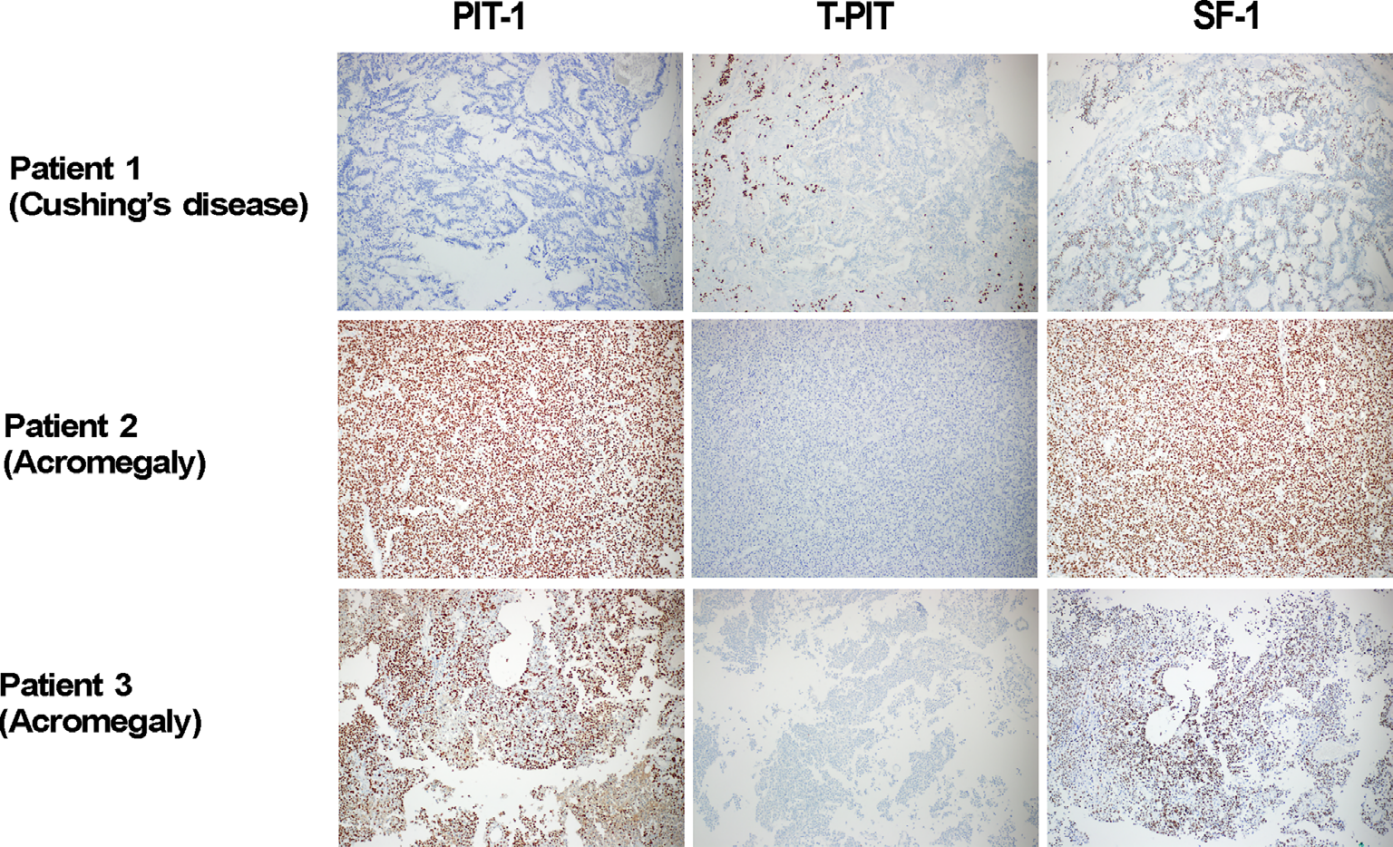
Three tumor cases with positive IHC for multiple transcriptions factors. In our prospective investigation on 484 pituitary adenomas, three unusual cases were identified as their cell lineages were not clearly classified. They were 1 Cushing’s disease patient with positive IHC for T-PIT, and SF-1, and two acromegalic patients with positive IHC for both PIT-1 and SF-1. In Patient 2, positive stains for PIT-1 and SF-1 were observed in the same cells. PIT-1, T-PIT and SF-1 X 100. IHC, immunohistochemistry; PIT-1, pituitary specific transcription factor 1; SF-1, steroidogenic factor 1; T-PIT, pituitary cell restricted factor.

First, we identified three unusual tumors that could not be classified into any specific category. These tumors were positive for multiple TFs; one Cushing’s disease patient had positive IHC for T-PIT, and SF-1, and two acromegalic patients had positive IHC for both PIT-1 and SF-1 (**Figure 2**). Complete resection was done in all three cases, but one acromegalic patient did not achieve endocrinological remission.

**Figure 3:**
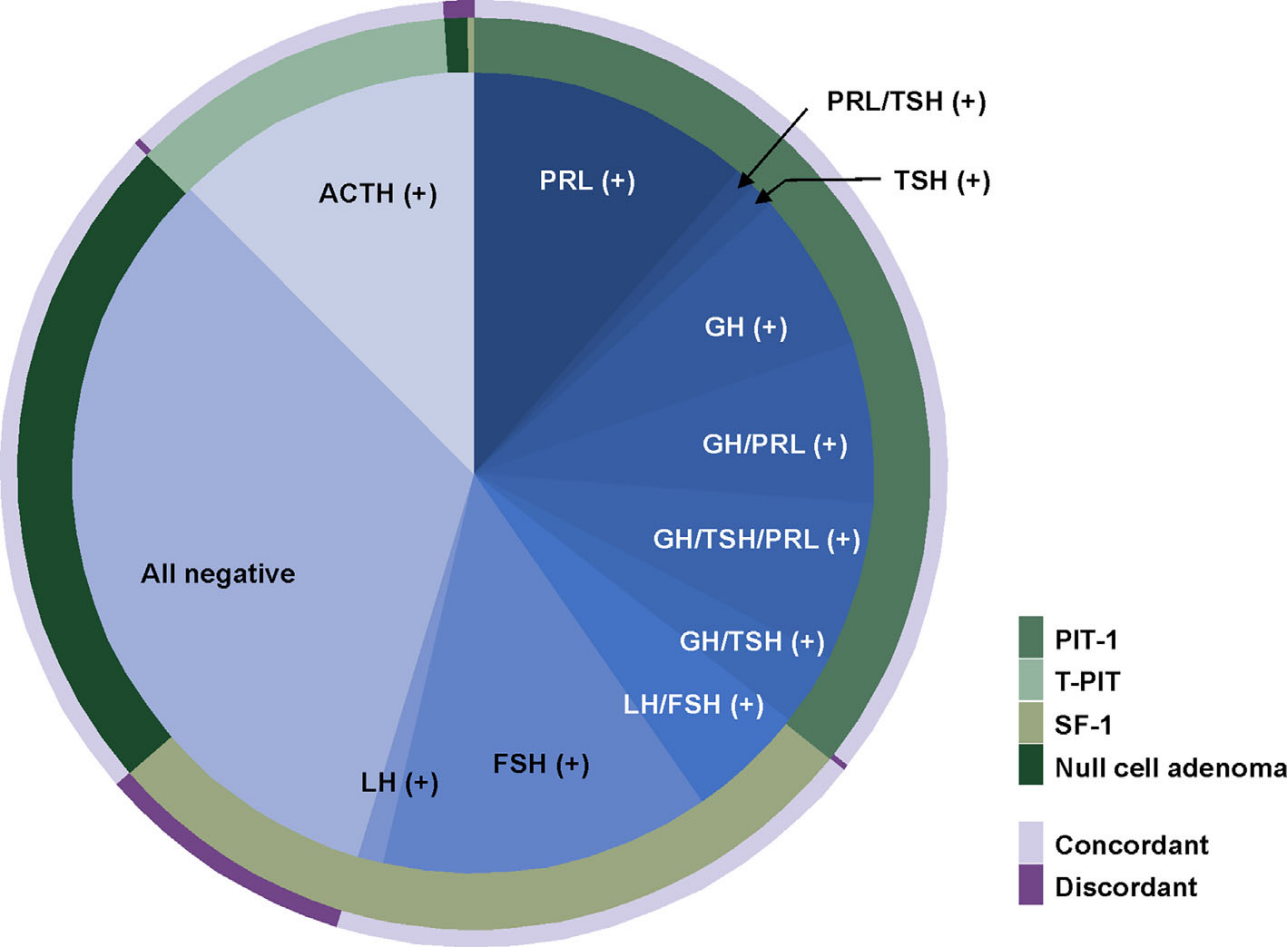
Comparison between immunohistochemical stain for transcription factors and pituitary hormones. The most common subtype was PIT-1 positive adenomas. In 171 PIT-1 positive adenomas, the most prevalent subtype was a prolactinoma followed by GH-positive tumors. The most common subtype of gonadotroph adenomas was a FSH-positive tumor and LH-positive tumors were the least common. Overall, 10.4% of cases showed discordance between TF stains and pituitary hormone stains. The majority of mismatched cases were gonadotroph adenomas of which stains were positive for SF-1 but negative for both FSH and LH. FSH, follicle stimulating hormone; GH, growth hormone; LH, luteinizing hormone; PIT-1, pituitary specific transcription factor 1.

For the other 481 patients, we evaluated whether their TF stains were well matched with their pituitary hormone stains (**Figure 3** and **Table 3**). The most common subtype was PIT-1 positive adenoma, and the overall matching rate was 89.6%. We identified 50 (10.4%) patients with pituitary adenomas whose pathological examinations were discrepant between TF and pituitary hormone stains. The majority of mismatched cases were gonadotroph adenomas (43 patients, 86.0%) with stains positive for SF-1 but negative for both FSH and LH. The second-most common tumor type was null cell adenomas positive for ACTH stain (n=4). The most common subtype of gonadotroph adenoma was FSH positive, while LH-positive tumors were the least common. In 171 PIT-1 positive adenomas, the most prevalent subtype was GH-positive tumors followed by prolactinoma. Tumors with positive stains for two or more hormones were more prevalent than single hormone-positive tumors (53.2% *vs*. 46.8%). PRL, GH and TSH stain were positive in 121 (70.8%), 107 (62.6%) and 40 (23.4%) PIT-1 positive adenomas, respectively.

**Table 3:** Comparison between immunohistochemical stain for TFs and pituitary hormones.

TF stain	Pituitary hormone stain				
	GH, PRL, TSH positive	ACTH positive	FSH, LH positive	All negative	Total
PIT-1 positive	171		1*		172 (35.8%)
T-PIT positive		55		1*	56 (11.6%)
SF-1 positive		1*	91	43*	135 (28.1%)
All negative		4*		114	118 (24.5%)
Total	171	60	92	158	481

*Cases with discrepancy between TF and pituitary hormone stains.

ACTH, adrenocorticotropic hormone; FSH, follicle stimulating hormone; GH, growth hormone; LH, luteinizing hormone; PIT-1, pituitary specific transcription factor 1; PRL, prolactin; SF-1, steroidogenic factor 1; TF, transcription factor; T-PIT, pituitary cell restricted factor; TSH, thyroid stimulating hormone.

### Comparison Between Histological Diagnosis and Clinical Diagnosis

For the 431 patients with concordant results between TF and pituitary hormone stains, comparative analysis was performed for their histological and clinical diagnoses (**Table 4**).

**Table 4:** Comparison between clinical manifestations and histology for 431 patients with concordant immunohistochemical stains for TFs and pituitary hormones.

Clinical diagnosis	PIT-1 positive	T-PIT positive	SF-1 positive	All negative	Total
Cushing’s disease	*1	32		*1	34
Acromegaly	84				84
Prolactinoma	55			*2	57
TSHoma	2				2
Non-functioning adenoma	*29	*23	91	111	254
Total	171	57	91	114	431

*Cases with discrepancy between clinical manifestations and histology.

PIT-1, pituitary specific transcription factor 1; SF-1, steroidogenic factor 1; TF, transcription factor; T-PIT, pituitary cell restricted factor; TSH, thyroid stimulating hormone.

Among 177 endocrine-active tumors, 174 (98.3%) had concordant clinical and histological diagnoses. When we compared 32 patients with endocrine-active corticotroph adenoma (Cushing’s disease) with its counterpart (23 silent corticotroph adenomas), we observed that tumors were much larger in silent corticotroph adenoma patients than in Cushing’s disease patients (25.3 mm *vs.* 12.4 mm, *P*<0.001). However, there was no statistically significant difference between the incidence of cavernous sinus invasion and total tumor removal. In the comparison between patients with functioning PIT-1 adenomas (GH-secreting adenoma, prolactinoma, and thyrotropinoma) and silent PIT-1 adenoma patients, we failed to identify any of these.

**Figure 4:**
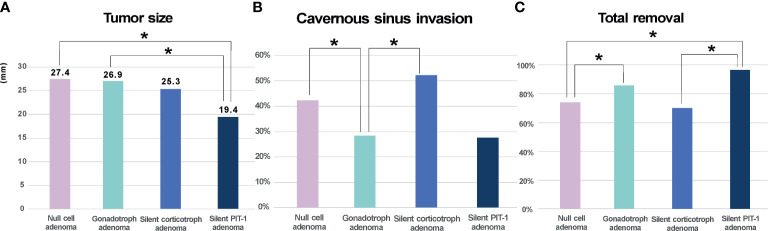
Clinical characteristics of endocrine-inactive tumors. **(A)**. Null cell adenomas and gonadotroph adenomas did not differ in size whereas silent PIT-1 adenomas were the smallest. **(B, C)**. Null cell adenomas showed more frequent cavernous sinus invasion than gonadotroph adenomas, which makes total removal loss feasible in patient with null cell adenomas. The incidence of cavernous sinus invasion was the highest in silent corticotroph adenoma. On the contrary, patients with gonadotroph adenomas were less likely to have cavernous sinus invasion. Among 29 patients with silent PIT-1 adenomas, 28 patients underwent total removal, which was significantly higher than patients with null cell adenomas or silent corticotroph adenomas. *Cavernous sinus invasion was identified by preoperative magnetic resonance imaging and based on surgeon’s inspection intraoperatively.* PIT-1, pituitary specific transcription factor 1; *P < 0.05.

A total of 254 endocrine-inactive tumors with no clinical or laboratory evidence of hormonal excess were further analyzed (**Figure 4**). In this group, there were more patients with true null cell adenomas (n=111) that were not positive for TFs or pituitary hormones than gonadotroph adenoma patients (n=91), followed by 23 patients with silent corticotroph adenoma and 29 with silent PIT-1 adenomas. Although tumor size did not differ, null cell adenomas showed more frequent cavernous sinus invasion than gonadotroph adenomas (*P*=0.043), which led to a difference in the likelihood of total tumor removal (*P*=0.039). Tumors were the smallest in patients with silent PIT-1 adenomas (*P*<0.001 *vs.* null cell adenomas and gonadotroph adenomas, *P*=0.054 *vs.* silent corticotroph adenomas). The incidence of cavernous sinus invasion was highest for silent corticotroph adenoma (*P*=0.032 *vs.* gonadotroph adenomas), which is a well-known aggressive form of pituitary adenoma. Conversely, patients with gonadotroph adenomas were less likely to have cavernous sinus invasion. Among 29 patients with silent PIT-1 adenomas, 28 underwent total removal, which was a significantly higher percentage than patients with null cell adenomas or silent corticotroph adenomas.

## Discussion

In recent decades, several TFs have been found to regulate cellular differentiation of the adenohypophysis, and they are also essential for differentiation and maturation of the neuroendocrine cells from Rathke’s pouch (2, 3). As TFs determine hormone-specific pituitary stem cell development, IHC for pituitary hormones and cell-specific TFs enables classification of differentiated pituitary adenomas based on pituitary cell lineage (4). Many studies have shown that TF staining can be a major ancillary diagnostic tool for more precise classification of pituitary adenomas (5–7). Considering that immunostain findings for pituitary hormones are often focal, very weak, or uncertain, TF staining may serve as a critical determinant for histological diagnoses in such instances. Based on these findings, the fourth edition of WHO classification system proposed a cell lineage-based classification scheme for pituitary adenomas, in which TFs such as PIT-1, T-PIT, and SF-1 serve as key classifiers. Many groups have adopted this new classification system and updated their guidelines for pathological diagnosis of pituitary adenomas (8). There have been several reports on early experiences with the new WHO classification system (9, 10). Before adopting a new classification system at our institution, we validated the reliability of antibodies for three TFs: PIT-1, T-PIT, and SF-1.

We created a cohort in which (1) patients’ pituitary hormone stains were all negative or singly positive and (2) their clinical manifestation and endocrine laboratory tests were consistent with the pituitary hormone stain results. In this 153-patient cohort, tumor hormonal activity was clearly defined by serum hormone levels and IHC for pituitary hormones. We then performed TF stains and evaluated whether the results were well matched to the previously established diagnosis. In this well-refined cohort, the diagnoses based on the new WHO classification were concordant with the original diagnosis. There were only five cases with discordant results, including three positive for SF-1 even though all negative hormonal stains were the majority. Although there has been concern that reliable commercial antibodies for T-PIT are not yet available (1), our T-PIT antibody was successfully validated for its accuracy (94.7%).

Convinced by the successful retrospective validation results, we changed our diagnostic protocol of pathological pituitary adenoma examination (**Figure 1**). We first performed IHC for three different TFs: PIT-1, T-PIT, and SF-1. After tumor cell lineage was identified, IHC was only performed for the relevant hormones. When the TF and pituitary hormone staining results were discordant, IHC was carried out for the remaining pituitary hormones. We experienced 3 cases with positive stains for multiple TFs; 2 in acromegaly and 1 in Cushing’s disease. We repeated IHC for both TFs and pituitary hormones and confirmed the same results. Moreover, these unusual cases have been reported by other groups (10–12). This should be further investigated as current WHO classification system does not provide how to classify theses unusual tumors. Recently, Neou et al. demonstrated that unsupervised multi-panel genomic classification of pituitary adenomas generally well correlates with cell lineage classification, which is good agreement with the new WHO classification scheme (13). For the cases with discrepancies between TFs and pituitary hormones, transcriptome or methylome analysis may help to clarify their identities.

While a guideline or consensus on the threshold of hormone-positive tumor cells for immunohistochemical classification is not available, it has been our strategy that only one positive, unequivocally neoplastic cell is regarded as significant. In our clinical practice, we have often experienced cases in which the presence of single hormone-positive tumor cell was well matched with clinical diagnosis. However, low threshold of positive stain for pituitary hormones in our institution may possibly result in different observations from the literature. Indeed, we have more tumors with positive stains for two or more hormones than single hormone-positive tumors in PIT-1 positive adenomas, and low threshold of hormone-positivity may be the reason for this observation.

In our earlier series (14) when TF stains were not available, 66.3% of clinically endocrine-inactive pituitary adenomas were negative for any of pituitary hormone stains and thus classified as null cell adenomas. In our current study, among 254 endocrine-inactive tumors with no clinical or laboratory evidence of hormonal excess, we identified 111 tumors (43.7%) true null cell adenomas. Although the proportion of null cell adenomas were much lowered, this is still much higher proportion compared with other groups (1, 9, 15, 16). One of the possible explanation for the discrepancies in the incidence of null cell adenomas is that gonadotroph adenomas are possibly underdiagnosed in our study although we validated the reliability of SF-1 antibody in our retrospective investigation. Among 50 patients with pituitary adenomas whose pathological examinations were discrepant between transcription factor stains and pituitary hormone stains, the majority of mismatched cases were gonadotroph adenomas (43 patients, 86.0%) with stains positive for SF-1 but negative for both FSH and LH. Considering the threshold of hormone-positivity is very low in our study, we believe it should be further validated whether single SF-1immunostain is sufficient to characterize gonadotroph adenomas and whether current antibody for SF-1 is a reliable. Further investigation with IHC for GATA2, GATA3 and alpha subunit would help the differentiation between true null cell adenomas and gonadotroph adenomas and thus provide the true prevalence of null cell adenomas (17–19). Unlike other endocrine-active adenomas, most gonadotroph adenomas are clinically non-functioning tumors that lack hormone overproduction. Although their cell linages are apparently different, distinguishing between gonadotroph and null cell adenomas has always been difficult. Traditionally, null cell adenomas were considered a synonym of pituitary hormone-negative pituitary adenomas; however, the new WHO classification clearly defined null adenomas as tumors that do not exhibit immunoreactivity for pituitary hormones or TFs. Nishioka et al. demonstrated that up to 95% of pituitary adenomas negative for any pituitary hormones actually expressed lineage-specific TFs: SF-1 and/or estrogen receptor-α positive in 67%, T-PIT positive in 27% and PIT-1 positive in 2% (15). Thus, they suggested only 5% of tumors were true null cell adenomas. This observation was supported by Mete et al., who reported that the incidence of null cell tumors in their series was only 4.5% (20). We identified 111 null cell adenomas out of 158 adenomas with negative stains for any pituitary hormones, suggesting that its prevalence may be much higher than previously reported (**Table 3**).

In the subgroup analysis on 254 clinically endocrine-inactive tumors (**Table 4**), null cell adenomas were the majority, followed by gonadotroph adenomas. We also observed more silent PIT-1 adenomas (n=29) than silent corticotroph adenomas (n=23), which was different from a previous observation (15). We compared these four subtypes of endocrine-inactive tumors in terms of size, cavernous invasion probability, and likelihood of total resection. Unsurprisingly, silent corticotroph adenomas showed the highest incidence of cavernous sinus invasion and the lowest possibility of complete tumor removal. Null cell adenomas, gonadotroph adenomas, and silent corticotroph adenomas were similar in size. Null cell adenomas were more likely to invade the cavernous sinus compared with gonadotroph adenomas. Consequently, total resection was less likely for null cell adenomas. Although this finding should be further validated with long-term follow-up (21), null adenomas seem to be clinically more aggressive than gonadotroph adenomas, suggesting that this discrimination may be critical for patient management.

## Conclusion

The new WHO classification scheme is mostly well matched with the traditional classification scheme. Gonadotroph adenomas lacking FSH and LH stains account for the majority of discrepancies in clinical settings, and further validation and characterization of this small subset of pituitary adenomas may be necessary. Null cell adenomas may be more prevalent than previously reported, which requires further verification. Until the new classification is further validated and interpreted with long-term clinical outcomes, routine histological examination should include a full slate of immunostains for both pituitary hormones and TFs.

## Data Availability Statement

The raw data supporting the conclusions of this article will be made available by the authors, without undue reservation.

## Ethics Statement

The studies involving human participants were reviewed and approved by Institutional Review Board of Severance Hospital. Written informed consent for participation was not required for this study in accordance with the national legislation and the institutional requirements.

## Author Contributions

Conceptualization, EK. Methodology, CK and EK. Software, EK. Validation, EK. Formal Analysis, SH, CK and EK. Investigation, SH, SeK, SL, EL, SuK, CK and EK. Resources, CK and EK. Data Curation, SH, CK and EK. Writing – Original Draft Preparation, SH. Writing – Review & Editing, EK. Visualization, EK. Supervision, EK. Project Administration, EK. Funding Acquisition, Not available. All authors contributed to the article and approved the submitted version.

## Funding

This study was supported by a faculty research grant of Yonsei University College of Medicine (6-2020-0224).

## Conflict of Interest

The authors declare that the research was conducted in the absence of any commercial or financial relationships that could be construed as a potential conflict of interest.

## Publisher’s Note

All claims expressed in this article are solely those of the authors and do not necessarily represent those of their affiliated organizations, or those of the publisher, the editors and the reviewers. Any product that may be evaluated in this article, or claim that may be made by its manufacturer, is not guaranteed or endorsed by the publisher.
